# Supplementary material for: Forewarned Is Forearmed: Machine Learning Algorithms for the Prediction of Catheter-Induced Coronary and Aortic Injuries
Source: Int J Environ Res Public Health. 2022 Dec 18;19(24):17002. doi: 10.3390/ijerph192417002 (PMC9779019; doi:10.3390/ijerph192417002)
Supplement: Supplementary file 1 [file ijerph-19-17002-s001.zip › ijerph-2107274-supplementary.pdf]

International Journal of
Environmental Research and Public Health

MDPI

Supplementary Materials

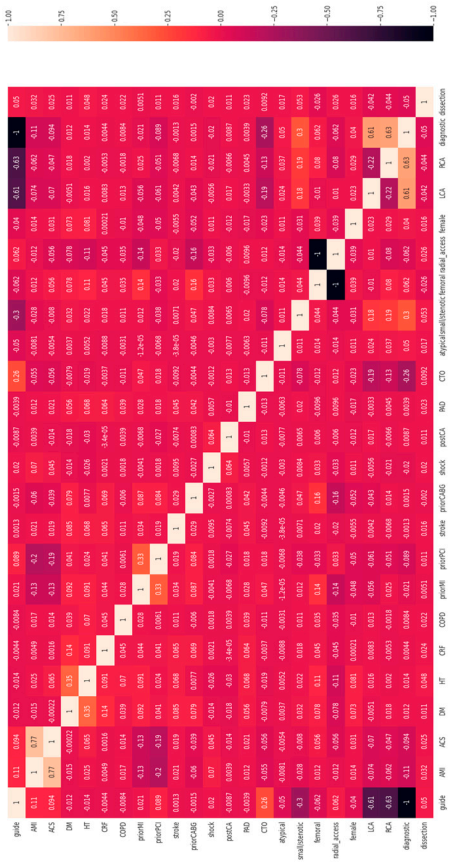

Figure S1. Correlation matrix of candidate variables (for abbreviations see Table S1).

Table S1. Univariate analysis of candidate variables.

	Feature	Odds_Ratio	*p* Value
**0**	guide	7.486	<0.001
**1**	AMI	3.246	<0.001
**2**	ACS	2.726	<0.001
**3**	DM	1.649	0.029
**4**	HT	4.982	<0.001
**5**	CRF	3.639	<0.001
**6**	COPD	4.404	<0.001
**7**	priorMI	1.246	0.303
**8**	priorPCI	1.543	0.029
**9**	stroke	2.993	0.005
**10**	priorCABG	0.845	0.846
**11**	shock	4.585	0.003
**12**	postCA	2.745	0.041
**13**	PAD	4.487	<0.001
**14**	CTO	1.890	0.100
**15**	atypical	4.987	0.010

**16**	small/stenotic	5.528	<0.001
**17**	femoral access	0.328	<0.001
**18**	radial access	3.045	<0.001
**19**	female	1.792	0.002
**20**	LCA	0.244	<0.001
**21**	RCA	0.234	<0.001
**22**	diagnostic	0.134	<0.001

ACS, acute coronary syndrome; AMI, acute myocardial infarction; atypical, atypical origin of coronary artery; CA, cardiac arrest; CABG, coronary artery bypass grafting; COPD, chronic obstructive pulmonary disease; CRF, chronic renal failure; CTO, chronic total occlusion procedure; diagnostic, angiography procedure; DM, diabetes mellitus; guide, use of a guiding catheter (angioplasty); HT, arterial hypertension; LCA, left coronary artery intubation; MI, myocardial infarction; PAD, peripheral arterial disease; PCI, percutaneous coronary intervention; RCA, right coronary artery intubation; small/stenotic, coronary ostium ≤ 3mm and/or with ≥ 30% stenosis.
